# Clinical significance of TRIM29 expression in patients with gastric cancer 

**Published:** 2022

**Authors:** Javad Farhadi, Jamshid Mehrzad, Hassan Mehrad-Majd, Alireza Motavalizadehkakhky

**Affiliations:** 1 *Department of Biochemistry, Neyshabur Branch, Islamic Azad University, Neyshabur, Iran*; 2 * Cancer Molecular Pathology Research Center, Mashhad University of Medical Sciences, Mashhad, Iran*; 3 *Department of Chemistry, Neyshabur Branch, Islamic Azad University, Neyshabur, Iran*

**Keywords:** Gastric Cancer, TRIM29, Β-Catenin, Cyclin D, Bcl-2, Survival, Prognosis

## Abstract

**Aim:**

The present study aimed to evaluate the expression profile, prognostic value, and possible correlation of TRIM29 with β-catenin, Cyclin D, and Bcl2 in Iranian patients with GC.

**Background:**

Tripartite Motif Containing 29 (TRIM29) has been reported to function as an oncogene or a tumor suppressor depending on the tumor type. This contextual function has created a controversial situation that needs to be fully delineated in various cancers. Although few studies have reported an elevated TRIM29 expression in gastric cancer (GC), its clinicopathological and prognostic values as well as possible molecular mechanisms are yet to be re-evaluated in different populations.

**Methods:**

Real-time quantitative PCR was used to detect TRIM29, β-catenin, Cyclin D, and Bcl-2 expression in 40 GC and their adjacent normal tissues. Patients were further stratified into high and low expression subgroups based on their TRIM29 expression levels. The association of TRIM29 expression level with β-catenin, Cyclin D, BCL2, some clinicopathological features, and patients' overall survival (OS) was assessed using appropriate statistical analyses.

**Results:**

The results showed a significantly higher TRIM29 expression level in GC tissues compared with their corresponding normal tissues (fold change=2.94, *p*=0.003). Patients with high TRIM29 expression levels exhibited poorer OS (HR=1.25, 95% CI: 1.06-1.47, *p*=0.007). High expression of TRIM29 was also associated with increased levels of β-catenin, Cyclin D, and Bcl-2 genes expression.

**Conclusion:**

Overexpression of TRIM29 is associated with poor prognosis in patients with GC and is probably mediated through β-catenin/Cyclin D/Bcl2 pathway and can be considered as a potential independent prognostic marker.

## Introduction

 Gastric cancer (GC) is the most prevalent malignancy in the upper gastrointestinal tract (1) and is responsible for over 768,000 annual deaths, according to the GLOBOCAN 2020 database (2). Patients with GC exhibit an average 5-year survival rate of <30% with remarkable regional variations in Asian, Eastern European, and South American countries (2). 

Despite substantial improvements in GC therapy, including surgery, radiation, chemotherapy, targeted therapy, and reduced mortality rate over the last decade, the disease burden is still considerable because of rapid tumor metastasis and high recurrence rates (3). Moreover, as GC is usually asymptomatic in the early stages and because of the lack of appropriate predictive markers, most GC patients are diagnosed at advanced stages, leading to a remarkably unsatisfactory prognosis (4, 5). Therefore, predicting clinical prognosis, especially in early stages, plays a critical role in reducing disease burden and improving overall survival. 

Biomarkers are one of the most valuable promising tools for the early detection and prognosis of different cancers. It is suggested that a patient’s stratification based on specific clinical biomarkers at the time of diagnosis could help in applying a treatment strategy compatible with the patient’s characteristics (6-8). To date, several prognostic biomarkers have been proposed to risk stratify GC patients. HER2 (9), FGFR2 (10), E-cadherin (11), the PI3K signaling axis (12), VEGF (13), and p53 (14) are among the most important prognostic markers that have been introduced for GC. However, the controversial prognostic values and lack of validation evidence of these biomarkers for clinical use has led to ongoing research to identify novel, effective, and more precise biomarkers.

The tripartite motif (TRIM) protein family is characterized by its unique structure containing three conserved domains, including one RING finger and two B-boxes, followed by a coiled-coil (RBCC) region (15-16). In recent years, abnormalities in many members of this protein family have been widely described in various human cancers (17-21). They are reported to contribute to various biological processes, namely cell proliferation, differentiation, apoptosis, migration, and oncogenesis (15, 17). Tripartite motif-containing 29 (TRIM29), also known as ATDC, is a member of the TRIM protein family and plays an essential role in regulating a wide variety of biological processes such as innate immune response (22). TRIM29 has also been reported to function as an oncogene or a tumor suppressor depending on the tumor type. While TRIM29 seems to serve as a tumor-suppressive factor in breast cancer and hepatocellular carcinoma (23, 24), it has shown an oncogenic feature in other types of malignancies such as ovarian (25) and cervical cancers (26). This contextual function has created a controversial situation that needs to be fully delineated in various cancers. It seems necessary to re-evaluate TRIM29 expression patterns and its probable molecular functions based on cancer type and patient’s ethnic background.

TRIM29 has been reported to be upregulated and associated with poor clinical outcomes in patients with GG (27-30). Overexpression of TRIM29 in GC tissues may result from different cellular contexts and molecular signaling pathways. As genetics and ethnicity are associated with GC incidence and mortality, the present study has aimed to re-evaluate the clinicopathological and prognostic values as well as the possible mechanism(s) through which this gene might be involved in the pathogenesis of GC in a group of Iranian patients. The TRIM29 expression pattern, its role in patients’ overall survival, and its correlation with expression levels of representative genes (Cyclin D, Bcl-2, and β-catenin) in other biological pathways in GC (cell cycle, apoptosis and Wnt signaling, respectively) were determined in this study. 

## Methods

**Patients Sample Collection**

Forty patients diagnosed with gastric cancer and having a median age of 65.73 ± 10.19 years who referred to the first affiliated hospital of Mashhad University of Medical Sciences, Mashhad, Iran, were enrolled in the present study between November 2015 and February 2018. Patients were selected if they met the following criteria: I) having GC relevant clinical symptoms (dysphagia, dyspepsia, gastrointestinal bleeding, anemia, weight loss, anorexia, inappetence, nausea, reflux, and vomiting); II) having no history of chemotherapy, radiotherapy, or surgery for GC; and III) confirmation of GC by pathology tests. Among them were 21 patients aged below 65 years (52.5%) and 19 patients aged over 65 years (47.5%). Moreover, 77.5% of participants were male (31 out of 40) and 22.5% were female (9 out of 40). The clinicopathologic features of the patients are summarized in Table 1. 

**Table 1:** Demographic features of patients

Clinico-pathologic features	Percentage (%)	
Gender	Male	Female
	77.5	22.5
Anemia	Positive	Negative
	67.5	32.5
Weight loss	Positive	Negative
	80	20
Bleeding	Positive	Negative
	37.5	62.5
Stomachache	Positive	Negative
	72.5	27.5
Vomiting and Nausea	Positive	Negative
	70	30
Reflux	Positive	Negative
	52.5	47.5
Anorexia	Positive	Negative
	37.5	62.5
Smocking	Positive	Negative
	45	55

Using endoscopy, specimens of both GC tissue and adjacent normal tissues (>5 cm away from tumor sites) were collected from each patient. All tissue specimens were kept in RNAlater for RNA preservation (Thermo Fisher Scientific, Waltham, MA, USA) at 4 °C overnight and then stored at -80 °C until RNA extraction. This study was conducted according to the Declaration of Helsinki protocol, and all participants provided signed consent before participating in this study. Moreover, the Ethics Committee of Mashhad University of Medical Sciences approved the study (IR.MUMS.MEDICAL.REC.1398.606).

**Table 2:** The list of the primers used in the present study

Gene	Forward primer	Reverse primer	Length (bp)
β-actin	5'-CACGAAACTACCTTCAACTCC-3'	5'-CATACTCCTGCTTGCTGATC-3'	265
TRIM29	5'-GCACCGGACACCATGAAGA-3'	5'-GGAGACGAGGGCTGGTATGA-3'	80
β-catenin	5'-TCTGAGGACAAGCCACAAGATTACA-3'	5'-TGGGCACCAATATCAAGTCCAA-3'	122
Cyclin D	5'-TGGAGGTCTGCGAGGAACA-3'	5'-TCATCTTAGAGGCCACGAACAT-3'	147
Bcl-2	5'-ACGGTGGTGGAGGAGCTCTT-3'	5'-CGGTTGACGCTCTCCACAC-3'	98

**Gene expression analysis using qRT-PCR**

After the tissues were processed, RNA was extracted from the samples using Trizol Reagent (Sangon Biotech Co., Ltd., Shanghai, China). The quality and the quantity of the extracted RNA were assessed by Nanodrop ND-1000 instrument (Thermo-Fisher Scientific, Waltham, MA, USA). Those RNAs whose optical density (OD) 260/280 nm ratio was more than 1.8 were stored for further analysis. Next, 2 µg of extracted RNA was subjected to reverse transcription reaction using a cDNA synthesis kit (Wizbiosolutions, Seongnam, Gyeonggi, Korea). The synthesized cDNA together with the reverse and forward primer of each gene (Table 2), SYBR Green master mix, and nuclease-free water were located at a light cycler instrument (Roche LightCycler® 96 System) to conduct the quantitative real-time PCR (qRT-PCR) analysis. The condition for thermal cycling was initial activation step of 600 s at 95 °C, 40 cycles of amplification (15 s at 95 °C, 30 s at 59 °C and 30 s at 72 °C), and termination step of 10 s at 95 °C, 60 s at 65 °C, and 1 s at 97 °C). Β-actin was used as the housekeeping gene. All tests were done in triplicate, and fold changes in each mRNA expression were calculated using 2^-ΔΔCt^.

**Statistical analysis**

All statistical analyses were performed using IBM SPSS version 22 for Windows (SPSS Inc., Chicago, IL, USA). The normality of continuous data was checked by one-sample Kolmogorov-Smirnov test. Continuous variables with normal distribution were presented as the mean ± standard deviation (SD); otherwise, they were given as median (IQR). The mRNA expression levels of TRIM29, β-catenin, Cyclin D, and BCL2 were compared between cancerous and normal tissues using the paired-sample t-test. Trim29 expression level was used to categorize all patients into two groups of high and low expression. The independent *t*, chi-square, and Fisher’s exact tests were applied to evaluate whether the expression of TRIM29 was associated with the clinicopathological parameters of the patients. Kaplan–Meier plot and Log Rank (Mantel-Cox) test were used to evaluate the patients' overall survival (OS). Cox proportional hazards regression in univariate and multivariate models were also applied to identify independent predictive factors for survival. A *p*-value <0.05 was considered as statistically significant. 

## Results

**TRIM29 expression was upregulated in cancerous compared to normal tissues**

**Figure 1:**
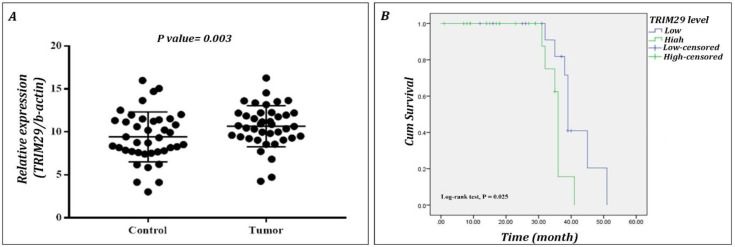
The expression of TRIM29 in GC tissue and its relation with overall survival (OS) of the patients. A) The qRT-PCR analysis results revealed a significant increased expression of TRIM29 in GC tissue compared to the normal tissue (P = 0.003).** B) **The results of the Kaplan-Meier analysis revealed that patients with high TRIM29 expression had a shorter OS than those with low TRIM29 (P = 0.025)

The current results showed a significantly increase in TRIM29 expression in GC tissues (*p*=0.003) (Fig. 1A). GC patients were classified according to their TRIM29 expression level as: higher level of TRIM29 named the high TRIM29 group (n=23); and lower TRIM29 expression known as the low TRIM29 group (n=17) (Table 3). Male patients showed a higher frequency of TRIM29 expression (91.3%) compared to female patients (41.2%) (*p*=0.015). However, no significant differences were found in clinicopathological features between the two mentioned groups (Table 3). 

**TRIM29 expression correlates with the overall survival of GC patients**

Kaplan–Meier analysis showed a lower overall survival (OS) in patients with higher expression of TRIM29 (36 months), while patients with lower expression had a prolonged OS (39 months) (*p*=0.025) (Fig. 1B). The results of uni- and multivariate Cox regression analyses regarding the effects of the interfering factors on TRIM29 expression and OS are presented in Table 4. Although univariate analysis revealed a correlation between TRIM29 expression and some patient characteristics, such as body weight (*p*=0.038), gender (*p*=0.044), bleeding (*p*=0.005), stomachache (*p*=0.014), anemia (*p*=0.009), and smoking (*p*=0.013), multivariate analysis revealed that only TRIM29 expression was correlated with OS (*p*=0.007). This indicates that the TRIM29 expression level could be an independent factor for poor prognosis in GC patients. 

**Table 3:** The relationship between TRIM29expression and clinicopathological features in GC patients

Variables	TRIM29 Expression			P-value
	High (n=23)	Low (n=17)		
Age (mean ± sd)*	66.83 ± 9.34	64.24 ± 11.38		0.434
Age Composition**	12 (52.2)	9 (52.9)	≤65	0.962
	11 (47.8)	8 (47.1)	>65	
Gender**	21 (91.3)	10 (58.8)	Male (%)	0.015
	2 (8.7)	7 (41.2)	Female (%)	
Anemia**	18 (78.3)	9 (52.9)	+	0.091
	5 (21.7)	8 (47.1)	-	
Weight loss**	20 (87.0)	12 (70.6)	+	0.250
	3 (13.0)	5 (29.4)	-	
Bleeding	11(47.8)	4(23.5)	Yes	0.117
	12(52.2)	13(76.5)	No	
Dysphagia***	1 (4.3)	2 (11.8)	No	0.577
	5 (21.7)	4 (23.5)	Rarely	
	17 (74.0)	11 (64.7)	Yes	
Tumor location***	10 (50.0)	8 (47.1)	Proximal	0.199
	5 (25.0)	8 (47.1)	Body	
	5 (25.0)	1 (5.9)	Distal	
Smoking**	11 (47.8)	7 (41.2)	Yes	0.676
	12 (52.2)	10 (58.8)	No	
Stomach ache**	16 (69.6)	13 (76.5)	Yes	0.730
	7 (30.4)	4 (23.5)	No	
Nausea**	18 (78.3)	10 (58.8)	Yes	0.185
	5 (21.7)	7 (41.2)	No	
Reflux**	13 (56.5)	8 (47.1)	Yes	0.554
	10 (43.5)	9 (52.9)	No	
Anorexia**	9 (39.1)	6 (35.3)	Yes	0.804
	14 (60.9)	11 (64.7)	No	

T-test (*), Chi-square test (**), and Fisher's exact test (***) were used to compare two groups

**Table 4:** Univariate and multivariate analyses for variables associated with the overall survival of GC patients

Variables	Univariate analysis		Multivariate analysis	
	HR (95 % CI)	*P*-value	HR (95 % CI)	*P*-value
TRIM29	1.22 (1.06-1.40)	0.005	1.25 (1.06-1.47)	0.007
Age	1.04 (0.99-1.08)	0.060		
Gender	0.29 (0.09-0.97)	0.044	1.93 (0.308-12.28)	0.485
Tumor location	1.54 (0.85-2.79)	0.154		
Anemia	0.012 (0.00-0.33)	0.009	0.001 (0.00-1.62)	0.927
Weight loss	0.02 (0.001-0.81)	0.038	0.001 (0.00-7.36)	0.941
Bleeding	0.31 (0.14-0.71)	0.005	0.81 (0.32-2.05)	0.660
Dysphagia	0.88 (0.66-1.16)	0.355		
Stomach ache	0.22 (0.06-0.74)	0.014	0.39 (0.09-1.53)	0.175
Smoking	0.34 (0.15-0.797)	0.013	1.23 (0.45-3.36)	0.689
Nausea and Vomiting	0.42 (0.16-1.14)	0.089		
Reflux	1.15 (0.52-2.54)	0.726		
Anorexia	0.55 (0.25-1.22)	0.140		

**Figure 2:**
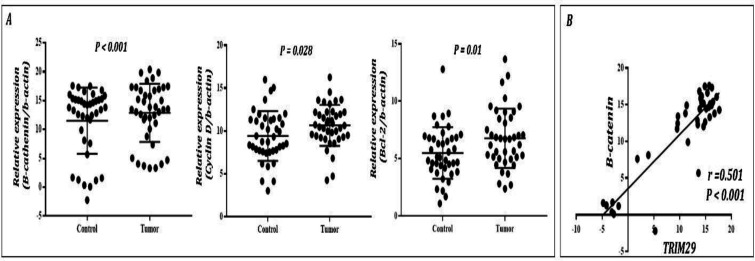
The expression of β-catenin, Cyclin D, and Bcl-2 in GC and normal tissues. A) As compared to the normal tissues, the results of the qRT-PCR analysis indicated that the expression of β-catenin, Cyclin D, and Bcl-2 remarkably increased in GC tissues. *P* < 0.05 is indicative of the statistical significance of the results. **B)** Pearson’s correlation analysis indicated that the expression of TRIM29 in GC tissues had a tight correlation with the expression of β-catenin (r=0.501; *P* < 0.001)

**The expression of TRIM29 is associated with β-catenin, Cyclin D, and Bcl-2 in GC patients**

In the current study, qRT-PCR analysis showed significantly elevated expression levels of cyclin D, Bcl-2, and β-catenin in GC tissue compared to the normal tissues (Fig. 2A). Pearson’s correlation test revealed a significant correlation between the expression of TRIM29 and β-catenin (r=0.51, *p*=0.001) (Fig. 2B), suggesting that the oncogenic activity of TRIM29 could be mediated through β-catenin signaling pathway.

## Discussion

Over the last decade, several members of the TRIM family of proteins have been shown to function as oncogenes or tumor suppressors. TRIM29 has been reported to have contextual function of tumor suppression or oncogenic depending on the tumor type. While TRIM29 acts as an oncogenic factor in most tumors, it plays a tumor suppressor role in other cancers. 

In the present study, we aimed to evaluate the expression of TRIM29 in GC patients. Our results showed that TRIM29 is overexpressed in GC and is correlated with shorter overall survival and poor clinical outcomes, confirming the oncogenic role of TRIM29 in gastric cancer. These results are in agreement with most previous reports on TRIM29 expression patterns in various cancers. A compelling body of evidence has suggested that TRIM29 might have oncogenic behavior in bladder (31), colorectal (32), and pancreatic (33) cancers. The difference in the behavior of a unique gene in the pathogenesis of cancers could be due to their interaction with diverse downstream targets. In lung cancer, TRIM29 has been reported to exert its oncogenic effects through activating autophagy flux (34). 

Overexpression of TRIM29 in gastric cancer has been reported to play an oncogenic role in cancer development and metastasis (27-30). Kosaka et al. reported that TRIM29 upregulation was associated with worse clinical outcomes, such as extent of tumor invasion, lymph node metastasis, larger tumor size, and poorer histological grade in GC patients (27). Qiu et al. also demonstrated an oncogenic function for TRIM29 overexpression in gastric cancer patients under the regulation of microRNA-185 (28). In addition, Wang et al. indicated that upregulation of TRIM29 in GC tissues was associated with tumor stage as well as lymph node and tumor-node-metastasis (TNM) stage (29). In line with these finding, the current results also strongly verified the overexpression of TRIM29 in GC tissues and its potential as a valuable prognostic biomarker for patient risk stratification.

The TRIM family is one of the main regulators of the Wnt signaling pathway that plays a fundamental role in regulating tumor metastasis and invasion (35, 36). Beside the crosstalk between the TRIM family and the Wnt signaling pathway, there is evidence that suggests that the Wnt signaling pathway plays a fundamental role in the pathogenesis of GC. Herein, we also found that the expression of β-catenin, a well-known component of Wnt axis, was elevated in GC tissues, and there was a significant correlation between the expression of this gene and TRIM29. In agreement with our findings, Zhou et al. have also indicated that the interaction between TRIM29 and β-catenin may be involved in lung cancer progression, probably through altering the expression of cyclin-dependent kinases (CDKs) (37). Cyclins are one of the main downstream targets of β-catenin. Cyclin D is notorious for its oncogenic role in the development of metastatic cancers (35). Apart from phosphorylating retinoblastoma (Rb) protein and driving G1 to S phase progression, Cyclin D prevents the degradation of anti-apoptotic proteins such as Bcl-2 (38). In agreement with the downregulation of TRIM29 in GC tissues, we also found remarkable upregulation in the expression of both Cyclin D and Bcl2. Although it is early to hazard a conjecture, it is reasonable to propose that the upregulation of TRIM29 in GC tissue probably stimulates the Wnt signaling axis which, in turn, reduces the survival and proliferative capacity of the malignant cells, at least partly, through the β-catenin/Cyclin D/Bcl2 axis. 

It should be noted that the current study has some limitations. Although the well-defined sampling strategy with an ethnically homogenous population is one of the main strengths of this study, the relatively small sample size and lack of a healthy control group may influence the strength of the results. It should also be noted that while the experiment was repeated three times with each gastric cancer tissue, insufficient data on patients’ clinicopathological features should be completed in subsequent studies. Finally, TRIM29 expression has only been considered in the mRNA level, while protein levels need to be evaluated using IHC and western blot analysis. 

This study suggests that overexpression of TRIM29 is associated with poor outcomes and shorter OS in patients with GC. This oncogenic function may be mediated through the Wnt/β-catenin/Cyclin D/Bcl2 signaling pathway. TRIM29 expression level has the potential to be considered as a prognostic marker in GC patients; however, further studies are suggested to ascertain its role in the pathogenesis and progression of GC.

## Conflict of interests

The authors declare that they have no conflict of interest.
